# Conjugated Linoleic Acid Isomers Affect Profile of Lipid Compounds and Intensity of Their Oxidation in Heart of Rats with Chemically-Induced Mammary Tumors—Preliminary Study

**DOI:** 10.3390/nu11092032

**Published:** 2019-08-30

**Authors:** Małgorzata Białek, Agnieszka Białek, Marian Czauderna

**Affiliations:** 1 The Kielanowski Institute of Animal Physiology and Nutrition, Polish Academy of Sciences, Instytucka 3, 05-110 Jabłonna, Poland; 2 Department of Bromatology, Medical University of Warsaw, Banacha 1, 02-097 Warsaw, Poland

**Keywords:** conjugated linoleic acid, heart, cancer, DMBA, fatty acids, oxysterols, rats

## Abstract

Breast cancer and cardiovascular diseases (CVD) have shared risk factors and mechanisms of pathogenicity, as proven by increased cardiac risk in breast cancer patients receiving anticancerogenic therapies and in cancer survivors. A growing mammary tumor may cause heart injury in cancer patients who have not yet been treated. This study aimed to evaluate the effect of conjugated linoleic acid (CLA) supplementation of female rats with 7,12-dimethylbenz(a)anthracene (DMBA)-induced cancerogenesis on fatty acids (FAs), conjugated FAs (CFAs), malondialdehyde (MDA), cholesterol and oxysterols content in cardiac tissue. FAs, cholesterol and oxysterols contents were determined by gas chromatography coupled with mass spectrometry, while the contents of CFAs and MDA were determined by high performance liquid chromatography with photodiode detection. Our results indicate that both CLA supplementation and the presence of tumors influence the lipid biomarkers of CVD. A significant interaction of both experimental factors was observed in the content of polyunsaturated FAs (PUFAs), n-6 PUFAs and CFAs. CLA supplementation significantly inhibited PUFA oxidation, as evidenced by the lower content of MDA in rats’ hearts, while the cancerous process intensified the oxidation of cholesterol, as confirmed by the elevated levels of 7-ketocholesterol in DMBA-treated rats. These results may significantly expand knowledge about CLA properties in terms of the prevention of co-existing non-communicable diseases.

## 1. Introduction

Cardiovascular diseases (CVD) and cancer are considered to be the major causes of death worldwide [1]. Annually, about 18 million of people die from CVD [2], while, in 2018, there were 18 million new cases of cancer and approximately 10 million cases of cancer deaths worldwide [1,3]. These disorders share not only this disgraceful leadership—they are also intrinsically linked via common risk factors, chronic inflammatory states and cardiac and vascular toxicities of chemo and radiotherapy [4,5,6]. Recently, Venneri et al. indicated that oncological patients who have not received any therapy may be endangered by CVD, as a growing mammary tumor may itself have a deleterious effect on the heart [7]. That is why cardio-oncology, based on the multidisciplinary analysis of patients’ needs, has been gaining popularity to improve existing preventive strategies and to standardize the processes of treatment and care [8,9].

Among the common risk factors of CVD and breast cancer, many are nutrition-related. The World Cancer Research Fund/American Institute of Cancer Research (WCRF/AICR) and American Heart Association (AHA) have given recommendations on dietary habits and healthy dietary patterns which, it is believed, may contribute to CVD and breast cancer prevention [10,11]. A beneficial plant-based diet with a high consumption of fruits, vegetables and whole grains is recommended, while the Western diet, rich in processed red meat and high-fat dairy products, is prohibited [11,12,13]. Restrictions of processed beef, whole-fat milk and dairy products consumption seem to be a double-edge sword, as those foodstuffs are the main dietary sources of conjugated linoleic acid (CLA) isomers—bioactive fatty acids (FAs) known for their multiple beneficial properties, e.g., anti-cancerogenic [14,15,16]. Due to the very low contents of these FAs in dietary sources [17], commercially available CLA supplements are gaining popularity for the maintenance of their adequate supply in the diet. The manufacturing of CLA supplements requires chemical synthesis from vegetable oil naturally rich in linoleic acid (*c*9*c*12C18:2; LA) via an alkaline isomerisation process [18].

CLA isomers are geometrical and positional isomers of LA in which two double bonds of *cis* (*c*) and *trans* (*t*) conformation are separated by a single bond. This slight difference in structure of the carbon chain results in a substantial change of their properties, e.g., an impact on tumor development. It was shown that dietary LA stimulates [19] but CLA suppress mammary carcinogenesis [20,21]. Because CLA isomers have also been suggested to exhibit antiatherogenic properties, it is anticipated that they may also affect the selected lipid biomarkers of cardiovascular disease in cardiac tissue. The effect of CLA isomers on the lipid profile of the hearts of laboratory animals in the physiological state have been studied [22,23,24], but little is known regarding the impact of these FAs on oxidative stress in cardiac tissue [25]. Moreover, lipid levels in tissues may also be influenced by coexisting diseases, e.g., tumors show a great affinity for polyunsaturated fatty acids (PUFAs) and may thus change the content of other FAs in nearby tissues [26]. Therefore, it is hypothesized that dietary CLA isomers administrated to animals with mammary tumors may reveal different impacts on selected CVD biomarkers than in healthy rats. Thus, this preliminary study was aimed on assessing whether dietary supplementation with commercial preparation containing CLA isomers influences the content of FAs, isomers of conjugated fatty acids (CFAs), and cholesterol in the hearts of female rats with chemically-induced mammary tumors. The impact of applied supplementation on the lipoperoxidation yield in cardiac tissue under cancer conditions was evaluated by the measurement of malondialdehyde (MDA) and oxysterols concentrations.

## 2. Materials and Methods 

### 2.1. Ethics Approval Statement 

This research and guiding principles in care and use of laboratory animals were approved by IInd Local Ethical Committee on Animal Experiments (No. 34/2008) at the Medical University of Warsaw. According to the 3R (replacement, reduction and refinement) ethical principle, the design of the study and experimental techniques used through the analysis allowed us to minimize the number of animals while maintaining a high statistical precision. There is no way to completely replace live animals with another research model in developmental programming experiments, especially in those concerning breast cancer development. Thus, Sprague Dawley female rats along with 7,12-dimethylbenz(a)anthracene as a cancerogenic agent have been chosen to model breast-cancer in humans because of several similarities in physiology, metabolism and pathology.

### 2.2. Dietary Ingredients

Laboratory fodder Labofeed H was purchased from the “Morawski” Feed and Concentrates Production Plant (Kcynia, Poland). Commercially available gel capsules of the Bio-C.L.A. dietary supplement, containing an equimolar mixture of *c*9*t*11CLA and *t*10*c*12CLA, as well as safflower oil (SAF oil), used as substrate for Bio-C.L.A. production, were kindly donated by Pharma Nord (Warsaw, Poland). Until the start of the experiment, they were stored according to the manufacturer’s recommendation. The detailed FA composition of dietary ingredients is presented in Table 1. 

### 2.3. Animal Experiment

**Figure 1:**
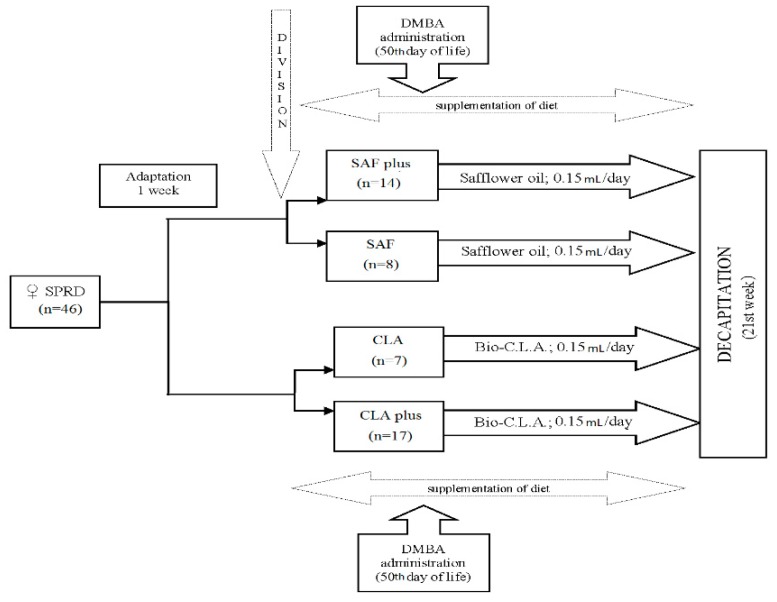
The scheme of experiment design. SPRD—Sprague Dawley rats; CLA—conjugated linoleic acids, DMBA—7,12-dimethylbenz(a)anthracene.

Forty-six female maiden Sprague Dawley rats (30 days old) were purchased from the Division of Experimental Animals, Department of General and Experimental Pathology (Medical University of Warsaw, Warsaw, Poland). They were housed in animal room at 21 °C in a 12 h light/12 h dark cycle, and they had access to the laboratory fodder and fresh drinking water ad libitum during the entire experiment. After an adaptation period (1 week), animals were randomly divided into 4 experimental groups. Beginning from the 37th day of life throughout the whole experiment, they received 0.15 mL/day either SAF oil (SAF and SAFplus group) or Bio-C.L.A. preparation (CLA and CLAplus groups) intragastrically. On the 50th day of life, each animal from the SAF plus and CLAplus groups received a single dose (80 mg/kg body weight) of 7,12-dimethylbenz(a)anthracene (DMBA, approximately 95%; Sigma-Aldrich, Saint Louis, MO, USA) for mammary tumors induction. Rats from SAF and the CLA groups were not treated with DMBA. The higher number of individuals in the DMBA-treated groups (SAFplus and CLAplus) was to ensure statistically adequate number of tumor-bearing rats in the case of unknown morbidity. Animals were monitored daily by an experienced veterinarian for specific signs of welfare and health disorders, and they were weighed weekly and palpated for the evaluation of tumor appearance. All the rats were decapitated in week 21 of the experiment, and after exsanguination, their hearts were excised, weighed and stored frozen in −80 °C for further analyses. A detailed scheme of experiment is presented on Figure 1.

### 2.4. Fatty Acids (Fas) and Conjugated Fatty Acids (Cfas) Profile in Hearts

Heart samples were subjected to alkaline hydrolysis prior to chromatographic analysis. The CFAs (dienes—CD; and trienes—CT) were analyzed directly (without derivatization) on a Waters HPLC 625LC system (Milford, MA, USA) equipped with four ion-exchange columns loaded with silver ions (Chromspher Lipids 5 μm, 250 × 4.6 mm; Varian, The Netherlands) and a photodiode array detector (PDA), with sorbic acid as the internal standard (IS) [27]. The total profile the of FAs was determined as methyl esters (FAME) with the addition of nonadecanoic acid (C19:0) as an IS on SHIMADZU GC-MS-QP2010 Plus EI gas chromatograph (GC) equipped with a quadruple mass selective detector Model 5973N (Tokyo, Japan) and a fused silica capillary-column BPX70 (120 m × 0.25 mm × 0.25 μm; Phenomenex, Torrance, CA, USA) [28].

On the basis of the FA profiles, indices attributed to the selected properties of FAs were also calculated according to the following equations [29,30,31]:

(1) Peroxidability index (*PI*)
  (1) $$\begin{array}{ll} PI= & \left(\%monoenoic\;FA\times 0.025\right)+\left(\%\;dienoic\;FA\times 1\right)+\;\left(\%\;trienoic\;FA\times 2\right)\\ & +\left(\%\;tetraenoic\;FA\times 3\right)+\;\left(\%\;pentaenoic\;FA\times 4\right)\;\\ & +\left(\%\;hexaenoic\;FA\times 5\right)\; \end{array}$$
(2) Index of atherogenicity (*AI*)
  (2) $$AI=\frac{C12:0+\left(4\times C14:0\right)+C16:0}{\sum^{\;}MUFA+\sum^{\;}n-6PUFA+\sum^{\;}n-3PUFA}$$
(3) Index of thrombogenicity (*TI*)
  (3) $$TI=\frac{C14:0+C16:0+C18:0}{\left(0.5\times \sum^{\;}MUFA\right)+\left(0.5\times \sum^{\;}n-6PUFA\right)+\left(3\times \sum^{\;}n-3PUFA\right)+\left(\frac{\sum^{\;}n-3PUFA}{\sum^{\;}n-6PUFA}\right)}$$
(4) Hypo/hypercholesterolemic index (*HH*)
  (4) $$HH=\frac{OA+LA+AA+ALA+EPA+DPA+DHA}{C14:0+C16:0}$$

### 2.5. Total Cholesterol and Oxysterols Content in Hearts

The contents of cholesterol and oxysterols were determined according to the slightly improved method of Czauderna et al. [32]. The improvements consisted of silylation with a BSTFA (*N*,*O*-Bis(trimethylsilyl)trifluoroacetamide, 99%; Sigma Aldrich, St. Louis, MO, USA) procedure. Derivatized analytes were separated on a GC-TOFMS Pegasus^®^ BT (LECO Corporation, St. Joseph, MI, USA) chromatograph equipped with a capillary column (30 m × 0.25 mm × 0.25 μm film thickness, Rxi^®^-17SilMS, Restek, Bellefonte, PA, USA). Identification was made on the basis of mass spectra and by a comparison of retention times of analytes with the following standards: cholesterol, 7α-hydroxycholesterol, 7β-hydroxycholesterol, cholesterol 5α,6α-epoxide, 7-ketocholesterol; Sigma, USA). For recoveries, 5α-cholestane (Sigma, St. Louis, MO, USA) as an IS was used.

### 2.6. Malondialdehyde (MDA) Concentration in Hearts

Prior to chromatographic analyses, hearts were subjected to a gentle alkaline saponification and derivatization with 2,4-dinitrophenylhydrazine (DNPH) followed by extraction with hexane [33]. The high-performance liquid chromatography UFLCXR system (SHIMADZU, Tokyo, Japan) equipped with a C18-column (Synergi 2.5 μm, Hydro-RP, 100 Å, 100 mm × 2 mm, Phenomenex, Torrance, CA) and a PDA operated in the UV range of 195–420 nm were used. A linear binary gradient of acetonitrile in water was used. MDA identification was based on the retention time and absorption UV spectrum (λ_max_ = 306 nm) of analytical standard (Sigma, St Louis, MO, USA). As an IS, a 1,5-pentanedialdehyde solution was used.

### 2.7. Statistical Analysis

The obtained results, presented as means ± standard deviation (SD), were elaborated with STATISTICA software (version 13, StatSoft Polska, Cracow, Poland) [34]. The normality of the data distribution was checked by the Shapiro–Wilk test. The effects of diet (D), presence of mammary tumors (MT) and interactions (D × MT) were evaluated using a two-way ANOVA. Only tumor-bearing rats from the SAFplus and CLAplus groups were considered in statistical analyses. When an interaction occurred (*p* ≤ 0.05), the significances of differences among groups were established using a post hoc honest significant difference (HSD) Tukey test for uneven numbers for variables with normal distribution or a multiple comparison test for variables with skew distribution (these data were log-transformed before statistical analyses). *p* ≤ 0.05 was considered significant. 

## 3. Results

As it is summarized in Table 1, in standard laboratory chow, the most prevalent FAs were LA and α-linolenic acid (ALA), while in SAF oil, *c*9C18:1, LA and C16:0 acids predominated. The chromatographic analysis of Bio-C.L.A. confirmed the manufacturer’s information that two main CLA isomers (*c*9*t*11C18:2 and *t*10*c*12C18:2) were present in equimolar amounts in this supplement, while SAF oil did not contain CFAs isomers. Among dietary ingredients, only Labofeed H contained cholesterol, while oxysterols were not detected in any of them.

No specific signs of welfare disorders (e.g., appetite loss, ruffling, sluggishness, apathy, hiding, and curling up) were observed in animals during the experiment, which confirmed the lack of the negative influence of the applied dietary supplementation. The influence of the experimental condition on the selected characteristics connected with carcinogenesis (partially published elsewhere [18,21]) is presented in Table 2. Throughout the experiment, there were no spontaneous tumors in animals not exposed to DMBA, regardless of the dietary modification. The administration of the carcinogenic agent to animals resulted in the presence of mammary gland tumors in the SAFplus and CLAplus groups, and these tumors were histopathologically identified as mammary adenocarcinomas [21].

DMBA administration significantly (*p* = 0.0000) increased the mass of hearts as well as the relative mass of hearts (measured in relation to the final body weight) in rats supplemented either with SAF oil or with Bio-C.L.A. (Table 2).

The total content of the assayed FAs was not affected by any of the applied experimental factors (D and MT) (Table 3). As far as saturated FAs (SFAs) are concerned, the administration of chemical carcinogen (DMBA) significantly lowered the content of caprylic acid (C8:0) in the group supplemented with SAF oil (*p* = 0.0000), while the content of heneicosanoic acid (C21:0) was significantly higher in the hearts of DMBA-treated rats irrespective of the type of supplement (*p* = 0.0302). The significant influence of both applied experimental factors as well as their interaction was shown in the case of lauric acid (C12:0). The interactions of both diet (D) and DMBA administration were also revealed in the content of pentadecanoic acid (C15:0) in the Bio-C.L.A.-supplemented group.

The sum of monounsaturated FAs (MUFA) was affected by the applied supplementation (*p* = 0.0198), particularly in animals without carcinogen administration. Moreover, an interactive action of both experimental factors was also exhibited (*p* = 0.0036). Similar changes were observed in the case of the predominant MUFA oleic acid (*c*9C18:1), of which amounts were significantly lower in the hearts of rats receiving the CLA-containing supplement (*p* = 0.0072). The amount of another positional isomer of the C18:1–*t*9C18:1 acid deposited in rats’ hearts was affected by diet (*p* = 0.0171) and tumors (*p* = 0.0005), as well as by their joint action (*p* = 0.0000). In animals fed with SFA oil, the cardiac content of *t*9C18:1 was decreased by DMBA administration, while in the Bio-C.L.A.-receiving rats, DMBA action was adverse. A significant increase in the *c*11C18:1 content in the hearts of tumor-bearing rats was also observed in the CLAplus group (Table 3).

In the total content of polyunsaturated FAs (PUFAs), n-6 PUFAs and docosapentaenoic acid (*c*7*c*10*c*13*c*16*c*19C22:5, DPA), a significant interaction of both experimental factors (D and MT) was observed (*p* = 0.0435, *p* = 0.0504 and *p* = 0.0001, respectively) (Table 3). The administration of the chemical carcinogen significantly influenced the content of *c*11*c*14C20:2 (*p* = 0.0005) and docosahexaenoic (*c4c7c10c13c16c19*C22:5, DHA) (*p* = 0.0433) acids. In the content of the two main CLA isomers (*c*9*t*11C18:2 and *t*10*c*12C18:2), the impact of diet was significantly exposed (*p* = 0.0000 and *p* = 0.0000, respectively). The amount of each of these isomers deposited in hearts of rats in the Bio-C.L.A.-obtaining groups was nearly equal but slightly higher in the cardiac tissue of the tumor-bearing animals (9.27 and 13.5 μg/g on average in the CLA and CLAplus groups, respectively). 

The significant impact of both applied experimental factors as well as their interactions were observed among all detected groups of conjugated fatty acid (CFAs) isomers except for the *cis,trans/trans,cis* (*ct/tc)* conjugated dienes (CD) and the *trans,trans,cis* (*ttc)* isomers of conjugated trienes (CT) (Table 4). The sum of CFAs in the hearts of animals supplemented with Bio-C.L.A. was several times higher than in the tissues of animals receiving SAF oil. The elevated content of CD, which was observed in the hearts of the CLA-supplemented groups, was revealed in animals both with (SAFplus vs CLAplus) and without (SAF vs CLA) tumors. The diminished levels of CT in cardiac tissue resulting from the presence of mammary tumors were more pronounced in the Bio-C.L.A.-supplemented groups (CLA vs CLAplus).

The content of malondialdehyde (MDA) was significantly affected by the diet fed to animals (*p* = 0.0012), as the amount of MDA in rats’ hearts was numerically lower in the Bio-C.L.A. fed groups (Table 5). The cholesterol and oxysterols concentrations in cardiac tissue of rats were not affected by any of applied experimental factor, with the exception of 7-ketocholesterol (7K), the levels of which were influenced by DMBA administration (*p* = 0.0139).

## 4. Discussion

Cancer is a chronic illness of which both internal (e.g., hormonal, immunological, and genetic) and environmental (e.g., dietary habits, physical activity, and radiation) factors play an important role in its development. However, some researchers have claimed that the genomic approach is nowadays overestimated, as lifestyle and acquired factors (potentially modifiable) account for 90–95% of disease burden [35]. The lesser influence of hereditary genetic factors on cancer development, together with the modifiable nature of the environmental factors seem to be sufficient prerequisites for considering cancer as a highly preventable disease [36]. Thirty-seven years since the National Cancer Act was signed by US President Richard Nixon, when the mortality rate for cancer was still extraordinarily high, the US Senate announced the 21st Century Cancer Access to Life-Saving Early detection, Research and Treatment (ALERT) Act, which is aimed at improving patient access to the prevention and early detection of cancer [37]. Scientists are still struggling to find the answer to the question why the ‘War on Cancer’ is still being lost while cancer is reportedly preventable?

The prevention of cancer may be carried out by primary and secondary actions or by their combination. Primary prevention, focused on the prohibition of carcinogenic agent effective contact with a target in the organism, may be implemented either by avoidance, the interruption of carcinogenic exposure, or by the reinforcement body defense through, e.g., dietary supplementation with chemopreventive agents [36]. Not every exposure of a carcinogen will necessarily elicit tumorigenesis, as the efficacy of this exposure is affected by, e.g., the dose and duration of exposure and the time of supplementation in relation to the carcinogen application. As far as breast cancer is concerned, it has been established that administration of CLA before the initiation of carcinogenesis and during the development of the mammary gland decreases the susceptibility of this tissue to the induction of neoplastic transformation. On the other hand, the exposure to CLA isomers after the activation of a carcinogen significantly reduces the number and growth of tumors [38]. As there is increased evidence that some specific dietary constituents exhibit anti-cancerogenic properties, not only before the carcinogenesis process starts but also afterwards [10], in our present experiment, CLA isomer supplementation occurred both before and after chemical carcinogen administration to animals (Figure 1).

In cancer patients and survivors, thrombocytopenia, hypercoagulability and coagulopathic states are common. Such comorbidities can specifically increase the risk of cardiovascular complications. Thanks to monitoring, early diagnosis and advances in the treatment of cancer, these patients will often die of CVD rather than the progression or recurrence of cancer [6].

The higher number of animals in DMBA-treated groups (SAFplus and CLAplus) ensured a statistically adequate number of tumor-bearing rats in the case of unknown morbidity. Applied dietary interventions did not influence the overall mass of the heart as well as the heart mass-to-body ratio, but the increase in these two parameters as a consequence of tumor presence was more pronounced in the rats supplemented with SAF oil (Table 2). This may indicate that animals deprived of CLA isomer supplementation may be more susceptible to cardiac hypertrophy after exposure to a chemical carcinogen. The lower body weight of tumor-bearing rats, exhibited to a greater extent in the SAF than in the CLA-fed groups, may be a first symptom of cancer cachexia. The significantly lower number of tumors which occurred in the Bio-C.L.A.-supplemented groups (*p* = 0.0326) in comparison to the rats fed SAF oil after DMBA administration seems to confirm the anticancerogenic properties of these bioactive CFA isomers (Table 2).

Though the preventive potential of CLA isomers has been confirmed, still little is known about the possible benefits and/or risks of CLA isomers on the heart in physiological conditions [39]. The existence of a pathological condition may significantly influence, e.g., FA demands (tumors selectively take up high amounts of polyunsaturated fatty acids (PUFAs)) [26]. Since FAs are not only important nutrients but also play a significant role in the composition of lipid bilayer membranes, it is likely that FA metabolism determines cell fate in a growing number of pathological conditions [40], especially tumorigenesis, which is connected with cell metastasis and angiogenesis [41].

The measurement of an FA profile, either of the whole heart or the cardiac sub-cellular membrane, was considered as complementary to the analysis of the structural and functional modification of the cardiovascular system of rats with a physiological condition [42,43]. Lipid biology in cardiac tissue is a rapidly growing field of research to establish the mechanisms by which the different lipid components of diets affect the cardiovascular system. Though novel FA derivatives (e.g., resolvins) and FA receptors (e.g., PPAR or Toll-like receptors) have been discovered, the role of different saturated and polyunsaturated FAs and their respective metabolites in cellular signal transduction and the possible implications for the development of cardiac tissue function is still being investigated [44]. In present study, mammary tumor elicitation was evoked by the administration of a single dose of DMBA. This diseased state, combined with the supplementation of animals’ diet with CLA isomers, is of utmost importance and a novel aspect of our work. To the authors’ best knowledge, this is the first attempt to implementat the cardio-oncological approach in a nutritional animal experiment. 

Though the commercial Bio-C.L.A. supplement contains a significant amount of two main CLA isomers (98.6 mg/g on average), only a very small amount of these CFAs was incorporated to the cardiac tissue of rats from the CLA (on average, 9.27 μg/g) and CLAplus (on average, 13.5 μg/g) groups. This may have resulted from the fact that heart contains a high amount of phospholipids (similarly to the liver and kidneys), while the CLA isomer is known to be mainly incorporated in triacylglycerols [24]. Some authors have hypothesized that the differences in the cellular metabolism yield of these CLA isomers being due to the content of the *t*10,*c*12 isomer tending to be lower in the liver, heart and kidneys than the *c*9,*t*11 homologue [24]. However, our results did not confirm this assumption, as in the cardiac tissue of CLA-supplemented rats, the content of the *t*10,*c*12 isomer was higher than the *c*9,*t*11 isomer, irrespective of the DMBA treatment (Table 3). However, in our previous research concerning serum [18] or the liver [45], predominating amounts of the *c*9,*t*11 isomer were observed, which could have resulted from the differences in metabolism of the *c*9,*t*11 and *t*10,*c*12 isomers, as some authors claim [46]. An adverse observation in case of cardiac tissue may suggest different intensities or different pathways of the CLA isomers’ metabolism in hearts. 

CFAs isomers were the compounds most affected by applied experimental condition (Table 4). Though the significantly higher contents of these FAs in the hearts of the CLA-supplemented groups were pretty obvious, the lower amounts of this group of FAs in both the DMBA-treated groups (SAFplus and CLAplus) in comparison to the untreated ones are interesting and should be pointed out. The results concerning CFAs are also worth emphasizing, because they seem to confirm our previous assumption of the ability of microbial population inhabiting the (monogastric) rats’ digestive tract to FA isomerization (unpublished data), as evidenced by the detection of other possible geometrical isomers (*tt* and *cc)* of CD differing from those delivered with the diet (*ct*/*tc)* (Table 4). Additionally, the conjugating activity/capability of rats was proven by the detection of CT isomers in the biological samples, though their dietary sources were used in present experiment. This activity may have resulted not only from the endogenous enzymes’ activity but also from the activity of microbiota inhabiting the cecum of rats, as CLA isomers exhibited a prebiotic effect [47]. This ability of CFA production seems to be connected with microorganisms specific for rats, as the gnotobiotic (germ-free) rats inoculated with microbiota obtained from the healthy humans were not supplied with CLA isomers [48]. The incorporation of various CLA metabolites (*cc, ct/tc, tt*) in the rats’ cardiac tissue may have resulted from the long-term supplementation (21 weeks) in our study, while the very short diet enrichment (six days) with CLA isomers did not cause such an effect [24]. Other possible explanations for this differences are the form/composition of the CLA applied into the animals’ diet (in our study, a commercial dietary supplement containing an equimolar mixture of two main CLA isomers vs an individual CLA isomers in the form of triacylglycerols) as well as animal strain (Sprague Dawley rats (SPRD) vs Wistar). Additionally, the gender of the animals (female vs male) may, to a considerable extent, have contributed to the observed discrepancies in CFA profiles, as was confirmed in earlier research in terms of heart rate, pressure and rate of contraction and relaxation [39].

An FA profile may be considered as a biomarker of CVD risk, as various groups of these compounds elicit different physiological effects, e.g., SFA is considered to pro-atherogenic and pro-thrombogenic, while PUFA is known as both an anti-atherogenic and anti-thrombogenic FA [29,30]. Due to DPA’s ability to interfere with cyclooxygenase (COX) and accelerate the lipooxygenase (LOX) pathways, this FA is considered to be the most potent inhibitor of collagen- or arachidonic acid (AA)-stimulated platelet aggregation among n-3 long chain PUFA [42]. Thus, the significant increase in the DPA content in the CLAplus group in comparison to the SAFplus group (Table 3) may indicate the cardio-protective effect of CLA supplementation in co-existing cancerous processes. Dietary CLA was also shown to suppress the formation of n-6 PUFA including dihomo γ-linolenic acid (DGLA) and AA in the physiological state (Table 3), which may presumably also affect eicosanoid biosynthesis [49].

It is well established, that increased FA oxidation in cardiac tissue, e.g., in case of diabetes is associated with cardiac dysfunction [44]. Peroxidability index, which reflects the peroxidation ability of tissue as a result of FA profile, did not differ among experimental groups (Table 3), which may ostensibly indicate lack of applied diet and/or cancerous process influence. However, this index reflects the state at the end of experiment and do not give any information on previous peroxidation intensity during experiment. Because of that we have decide to measure the levels of other biomarkers, which reflect not only FA peroxidation intensity (MDA) but also other lipid compounds (oxysterols). 

Elevated MDA levels, which is a strong mutagen, were observed in serum of patients suffering from breast and lung cancer [50]. In present experiment significantly higher content of MDA in the hearts of rats supplemented with SAF oil in comparison to the CLA-supplemented groups, irrespective of the presence of mammary tumors, may indicate the potential strong antioxidant activity of Bio-C.L.A. (Table 5). Similar properties were previously stated not only for single CLA isomers but also in combination with other bioactive compounds (e.g., Se) [51]. It may also suggest, that in this special animals model, CLA isomers, due to their antioxidant properties inhibit the activity of cytochrome P450 enzymes, and, as a consequence, diminish DMBA activation, which result in decrease breast cancer incidence in the CLAplus group. Another possibility is the fact that the auto-oxidation of CLA produced furan fatty acids which may protect against oxidant-mediated toxicity [52].

Recently, not only the products of PUFA oxidation but also oxysterols have gained popularity as useful biomarkers of oxidative stress. Oxysterols are a large family of lipid compounds and are one of plausible form of cholesterol transport, as well as intermediates in synthesis of bile acids and steroid hormones [53]. They have been arising endogenously during the enzymatic and non-enzymatic oxidation of cholesterol [54] or may exogenously enter the living organism with the food products where they were generated, e.g., during storage and/or processing [55]. Inasmuch as the dietary ingredients were not the exogenous source of oxysterols (Table 1), the whole detected amounts of these compounds originated from the oxidative transformation of the endogenous and supplied cholesterol. Oxysterols exert pleiotropic functions, as they may be biomarkers and/or the plausible cause of various pathological states (the pathophysiological biochemical activity of oxysterols may be even two times higher in comparison to cholesterol) [56]. The very high levels of oxysterols were found in hearts of hamsters fed a diet enriched with different doses of them, so these groups of oxidation products seem to exhibit a serious cardiac risk [57]. The concentration of 7K in present study was more than twenty orders of magnitude higher than content of 7β-hydroxycholesterol (7BOH) in the hearts of rats from the SAFplus group (2.84 ± 1.41 μg/g vs 0.14 ± 0.03 μg/g). This ratio in the hearts of the CLA-supplemented rats was lower, irrespective of the DMBA treatment (~13), which is a very promising observation because 7K—the most abundant non-enzymatically formed oxysterol—is mainly responsible for adverse health effects such as foam cell formation [55]. The elevated proportion of 7K and 7BOH that has also been reported in the cardiac tissue of chronically ethanol-fed rats [58] may indicate that heart is concomitantly affected in the course of many, seemingly unrelated, disorders. 

The content of cholesterol in the hearts of rats supplemented with 1% of an equimolar mixture of two main CLA isomers was much lower as compared to the hearts of animals which received 5% of individual CLA isomers in the form of triacylglycerols [24]. The results of the single CLA isomer in humans has shown that rumenic acid has a relatively beneficial influence, whereas *t*10,*c*12CLA seems to exert a proatherogenic effect as it decreases high density lipoproteins (HDL) and increases triacylgycerols (TAG) and very low density lipoproteins (VLDL) levels in obese male subjects [59]. An elevated CLA percentage share in the total FA pool in the serum of elderly men was adversely associated with cholesterol, high-density lipoprotein cholesterol, and reduced heart failure risk—but only for those with higher dairy fat intake as a major dietary source of CLA isomers (test for interaction, *p* = 0.03) [60]. Interestingly, CLA isomers given to female SPRD rats did not influence the cardiac function in contract to male SPRD rats, in which CLA isomers decreased heart rate, diastolic pressure, and systolic pressure [39].

The present study clearly shows that CLA supplementation can modify the FAs accumulated in cardiac tissue and significantly inhibit PUFA oxidation, while the cancerous process intensified the oxidation of cholesterol.

The main limitation of this study is that the obtained results may not be directly transferred into the humans. This is not only due to the differences in rat and human metabolism but also because the research material was hearts obtained post-mortem, the procedure for which is not applicable to humans, for both ethical and moral concerns. However, it should be emphasized that research conducted on an animal model allows for a proper and more accurate design of experiments involving people, and, as such, the results may indicate potential relationships and mechanisms that need to be verified. Moreover, this study may expand knowledge on CLA activity and may contribute to further cardio-oncological research with different types of cancer, different models, and diversified CLA doses.

## 5. Conclusions

Though the precise mechanism of how CLA isomer supplementation affects the heart in cases of DMBA exposure is still only anticipated, the results of present experiment may contribute to better explanations. Our results indicate that both CLA supplementation and the presence of mammary tumors influence the selected lipid biomarkers of CVDs. A significant interaction of both experimental factors was observed in the total content of PUFAs, n-6 PUFAs as well as CFAs. CLA supplementation significantly inhibited PUFA oxidation, as evidenced by the lower content of MDA, while the cancerous process intensified the oxidation of cholesterol, as confirmed by the elevated levels of 7K in the hearts of DMBA-treated rats. Our data may have important implications to possible functional and/or structural modifications in cardiac tissue. Moreover, the used research model may constitute a solid base on which to perform further research, e.g., to establish the influence of the dietary supplementation of CLA isomers during chemically-induced carcinogenesis on the functional and hemodynamic parameters of the heart.

## Figures and Tables

**Table 1:** Composition of dietary ingredients administered to rats.

Fatty Acid	Labofeed H	Safflower Oil	Bio-C.L.A.
**C6:0 (μg/g** **)**	10.4	nd	nd
**C8:0 (mg/g)**	nd	nd	1.23
**C10:0 (μg/g)**	nd	nd	917
**C12:0 (μg/g** **)**	4.8	nd	29.2
**C14:0 (μg/g)**	18.6	209	209
**C15:0 (μg/g)**	10.0	53.8	nd
**C16:0 (mg/g)**	1.05	13.9	16.2
***c7* C16:1 (μg/g)**	11.9	167	31.8
***c*9 C16:1 (μg/g)**	16.4	266	235
**C17:0 (μg/g)**	10.0	61.4	77.5
***c6*C17:1 (μg/g)**	6.2	nd	nd
***c*9C17:1 (μg/g)**	nd	69.0	54.2
**C18:0 (mg/g)**	0.44	0.01	6.48
***t*11C18:1 (μg/g)**	nd	nd	53.4
***c*9 C18:1 (mg/g)**	1.12	130	37.0
***c*11 C18:1 (mg/g)**	0.04	2.12	2.66
***t*9*c*12 C18:2 (μg/g)**	nd	nd	202
***c*9*c*12 C18:2 (LA) (mg/g)**	4.12	75.3	42.7
***c*9*c*12*c*15 C18:3 (ALA) (mg/g)**	2.21	0.95	0.00
**C20:0 (μg/g)**	10.5	957	820
***c*9*t*11C18:2 (mg/g)**	nd	nd	99.6
***t*7*c*9C18:2 (μg/g)**	nd	nd	944
***t*10*c*12C18:2 (mg/g)**	nd	nd	97.6
***c*11*c*13C18:2 (mg/g)**	nd	nd	4.13
***c*9*c*11C18:2 (μg/g)**	nd	nd	699
***c*11C20:1 (mg/g)**	10.4	619	nd
***c*8*c*11*c*14*c*17C20:4n-3 (μg/g)**	nd	672	nd
**C22:0 (μg/g)**	5.4	nd	359
**C24:0 (μg/g)**	nd	202	64.9
***c*15 C24:1 (μg/g)**	nd	240	187
**Conjugated fatty acids (mg/g)**			
**ƩCFA**	nd	0.49	192
**ƩCD**	nd	0.23	189
***tt* CD**	nd	0.17	5.18
***ct/tc* CD**	nd	0.05	178
***cc* CD**	nd	nd	6.23
**ƩCT:**	0.00	0.26	3.00
***ttt* CT**	nd	0.22	2.78
***ttc* CT**	nd	0.02	0.22
***cct* CT**	0.00	0.02	0.00
**Cholesterol (μg/g)**	155	nd	nd
**7AOH (μg/g)**	nd	nd	nd
**7BOH (μg/g)**	nd	nd	nd
**5,6AE (μg/g)**	nd	nd	nd
**7K (μg/g)**	nd	nd	nd
**MDA (ng/g)**	nd	nd	nd

LA—linoleic acid; ALA—α-linolenic acid; CFA—conjugated fatty acids; CD—conjugated dienes; CT—conjugated trienes; *cc —cis,cis* isomers; *ct/tc—cis,trans/ trans,cis* isomers; *tt—trans,trans* isomers; *ttt—trans,trans,trans* isomers; *ttc—trans,trans,cis* isomers; *cct—cis,cis,trans* isomers; MDA—malondialdehyde; 7AOH—7α-hydroxycholesterol; 7BOH—7β-hydroxycholesterol; 5,6AE—cholesterol 5α,6α-epoxide; 7K—7-ketocholesterol; nd—not detected.

**Table 2:** Characteristics of groups of rats supplemented with safflower oil (SAF oil) or Bio-C.L.A.

Diet		SAF Oil		Bio-C.L.A.		*p*-Values for Two-Way ANOVA		
	Group	SAF	SAFplus	CLA	CLAplus	Diet (D)	Mammary Tumors (MT)	Interaction
Variables								(D × MT)
**Total number of animals**		8	14	7	17	-	-	-
**Number of animals with tumors**		0	13	0	15	-	-	-
**Cancer incidence (%)**		0	93	0	88	-	-	-
**Final body weight (g)**		223 ± 10.9	212 ± 10.5	226 ± 15.8	223 ± 18.1	0.15	0.13	0.39
**Mass of heart (g)**		0.78 ± 0.05	0.94 ± 0.10	0.84 ± 0.08	0.95 ± 0.10	0.19	0	0.38
**Heart mass – body mass ratio (%)**		0.35 ± 0.02	0.44 ± 0.05	0.37 ± 0.02	0.43 ± 0.04	0.71	0	0.12
**First tumor appearance (week of life)**		0	18 ± 1.79	0	20 ± 1.64	0.26	0	0.26
**Total number of tumors**		0	63^c^	0^a^	28^b^	0.0326	0	0.0326
**Number of tumors per animal**		0	4.5	0	1.6	-	-	-
**Average tumor mass (g)**		0	5.4 ± 1.7	0	2.5 ± 0.4	0.0195	0.0017	0.19
**Tumor mass – body mass ratio (%)**		0	2.6	0	1.1	0.17	0.0008	0.17

Data are shown as mean values ± standard deviation (SD). *p-*value ≤ 0.05—significant differences among groups in the two-way ANOVA. 0.00—amount was below the quantification limit (<LOQ). D—diet; MT—mammary tumors, D × MT—interaction; When the interaction (D × MT) occurred, the significance of differences among groups was further analyzed by a post hoc HSD Tukey test or multiple comparison tests. ^a, b, c^—values with different superscripts in rows significantly differ at *p-*value ≤ 0.05. SAF group—animals receiving safflower oil; SAFplus group—animals receiving safflower oil treated with DMBA; CLA group—group of animals receiving Bio-C.L.A.; CLAplus group—group of animals receiving Bio-C.L.A. treated with DMBA.

**Table 3:** Profile of fatty acids (FAs) in hearts of rats supplemented with safflower oil (SAF oil) or Bio-C.L.A.

Diet		SAF Oil		Bio-C.L.A.		*p-*Values for Two-Way ANOVA		
	Group	SAF (n = 8)	SAFplus	CLA (n = 7)	CLAplus	Diet (D)	Mammary Tumors (MT)	Interaction
Variables			(n = 14)		(n = 17)			(D × MT)
**ƩFAs (mg/g)**		6.79 ± 19	6.42 ± 0.84	6.31 ± 0.74	6.73 ± 0.59	0.7	0.9	0.09
**C8:0 (μg/g)**		48.2 ± 17.0^b^	0.00 ± 0.00^a^	30.7 ± 12.5^ab^	23.7 ± 9.32^ab^	0.55	0	0
**C12:0 (μg/g)**		0.00 ± 0.00^a^	0.00 ± 0.00^a^	0.00 ± 0.00^a^	8.80 ± 3.95^b^	0	0	0
**C14:0 (μg/g)**		22.0 ± 4.95	16.9 ± 5.31	20.9 ± 6.32	19.6 ± 5.96	0.69	0.11	0.34
**C15:0 (μg/g)**		12.1 ± 2.80^ab^	11.7 ± 5.12^ab^	8.27 ± 3.19^a^	16.1 ± 7.42^b^	0.87	0.07	0.048
**C16:0 (μg/g)**		970 ± 44.4	927 ± 127	958 ± 61.0	963 ± 105	0.73	0.57	0.48
**C17:0 (μg/g)**		43.2 ± 6.10	39.3 ± 14.9	40.9 ± 10.3	40.9 ± 10.7	0.92	0.63	0.62
**C18:0 (mg/g)**		2.06 ± 0.18	2.00 ± 0.31	1.97 ± 0.16	1.95 ± 0.17	0.37	0.59	0.8
**C20:0 (μg/g)**		9.74 ± 5.18	7.13 ± 3.88	7.36 ± 3.13	10.5 ± 5.65	0.75	0.86	0.08
**C21:0 (μg/g)**		6.35 ± 3.29	10.7 ± 5.48	3.92 ± 2.53	7.66 ± 4.53	0.13	0.0302	0.86
**Ʃ SFA (mg/g)**		3.11 ± 0.16	3.00 ± 0.45	3.03 ± 0.23	3.03 ± 0.27	0.81	0.61	0.61
**A-SFA (μg/g)**		992 ± 46.4	943 ± 128	976 ± 64.5	985 ± 108	0.72	0.57	0.41
**T-SFA (mg/g)**		3.00 ± 0.16	2.94 ± 0.43	2.95 ± 0.23	2.93 ± 0.26	0.78	0.73	0.85
***c*7C16:1 (μg/g)**		12.2 ± 4.24	12.3 ± 4.58	11.3 ± 6.51	14.6 ± 8.26	0.75	0.46	0.49
***c*9C16:1 (μg/g)**		12.0 ± 4.63	12.4 ± 8.07	10.4 ± 2.61	13.3 ± 6.62	0.88	0.45	0.57
***t*9C18:1 (μg/g)**		16.1 ± 4.25^b^	0.00 ± 0.00^a^	8.23 ± 4.99^ab^	14.3 ± 4.24^b^	0.0171	0.0005	0
***c*9C18:1 (μg/g)**		269 ± 27.3^b^	199 ± 60.5^ab^	181 ± 37.9^a^	196 ± 43.6^ab^	0.0072	0.1	0.0109
***c*11C18:1 (μg/g)**		141 ± 6.89^ab^	131 ± 16.4^ab^	117 ± 19.5^a^	144 ± 14.3^b^	0.3	0.11	0.0012
**Ʃ MUFA (μg/g)**		449 ± 24.5^b^	356 ± 79.8^ab^	327 ± 62.1^a^	371 ± 62.0^ab^	0.0198	0.27	0.0036
**LA (mg/g)**		1.79 ± 0.89	1.67 ± 0.29	1.59 ± 0.28	1.80 ± 0.26	0.66	0.56	0.07
**ALA (μg/g)**		69.9 ± 11.5	27.9 ± 7.39	21.2 ± 8.16	32.9 ± 9.66	0.17	0.34	0.09
***c*9*t*11C18:2 (μg/g)**		0.00 ± 0.00	0.00 ± 0.00	9.15 ± 7.99	13.4 ± 6.32	0	0.21	0.21
***t*10*c*12C18:2 (μg/g)**		0.00 ± 0.00	0.00 ± 0.00	9.38 ± 6.19	13.6 ± 7.31	0	0.21	0.21
***c*11*c*14C20:2 (μg/g)**		6.49 ± 2.04	14.8 ± 7.92	7.20 ± 4.73	13.7 ± 4.74	0.93	0.0005	0.65
**DGLA (μg/g)**		17.8 ± 6.66	16.9 ± 5.51	12.3 ± 4.66	18.6 ± 6.38	0.33	0.18	0.07
**AA (μg/g)**		907 ± 58.0	851 ± 126	823 ± 107	892 ± 109	0.54	0.85	0.09
**EPA (μg/g)**		12.7 ± 10.6	12.1 ± 2.06	15.5 ± 8.03	14.3 ± 6.06	0.41	0.76	0.91
**DPA (μg/g)**		106 ± 4.77^abc^	92.6 ± 12.7^a^	94.3 ± 10.8^ab^	124 ± 20.2^c^	0.06	0.12	0.0001
**DHA (μg/g)**		248 ± 44.9	381 ± 61.4	264 ± 78.7	412 ± 49.3	0.22	0.0433	0.69
**Ʃ PUFA (mg/g)**		3.23 ± 0.16	3.06 ± 0.46	2.95 ± 0.47	3.33 ± 0.33	0.96	0.41	0.0435
**n-3 PUFA (μg/g)**		498 ± 47.0	505 ± 71.2	504 ± 109	583 ± 57.5	0.09	0.09	0.15
**n-6 PUFA (mg/g)**		2.73 ± 0.13	2.56 ± 0.41	2.43 ± 0.38	2.72 ± 0.31	0.56	0.6	0.0504
**Indices**								
**PI**		6892 ± 402	6629 ± 935	6447 ± 1038	7232 ± 653	0.77	0.34	0.06
**AI**		0.03 ± 0.00	0.03 ± 0.01	0.03 ± 0.01	0.03 ± 0.07	0.5	0.17	0.41
**TI**		0.96 ± 0.08^ab^	1.00 ± 0.14^ab^	1.04 ± 0.12^b^	0.89 ± 0.07^a^	0.64	0.13	0.013
**HH**		3.37 ± 0.44	3.44 ± 0.46	3.16 ± 0.34	3.55 ± 0.27	0.68	0.07	0.21

Data are shown as mean values ± standard deviation (SD). *p-*value ≤ 0.05—significant differences among groups in the two-way ANOVA. 0.00—amount was below the quantification limit (<LOQ). D—diet; MT—mammary tumors, D × MT—interaction; When the interaction (D × MT) occurred, the significance of differences among groups was further analyzed by a post hoc HSD Tukey test or multiple comparison tests. ^a, b, c^—values with different superscripts in rows significantly differ at *p-*value ≤ 0.05. SAF group—animals receiving safflower oil; SAFplus group—animals receiving safflower oil treated with DMBA; CLA group—group of animals receiving Bio-C.L.A.; CLAplus group—group of animals receiving Bio-C.L.A. treated with DMBA; FA—fatty acids; LA— linoleic acid; ALA—α-linolenic acid; AA—arachidonic acid; EPA—eicosapentaenoic acid; DPA—docosapentaenoic acid; DGLA—dihomo γ-linolenic acid; DHA—docosahexaenoic acid; SFA—saturated fatty acids; A-SFA—atherogenic saturated fatty acids; T-SFA—thrombogenic saturated fatty acids; MUFA—monounsaturated fatty acids; PUFA—polyunsaturated fatty acids; n-3 PUFA—n-3 polyunsaturated fatty acids; n-6 PUFA—n-6 polyunsaturated fatty acids; n-6/n-3—ratio of n-6 PUFA to n-3 PUFA; AI—atherogenicity index; TI—thrombogenicity index; HH—hypo-to-hyper-cholesterolemic FA ratio.

**Table 4:** Profile of conjugated fatty acids (CFAs) in hearts of rats supplemented with safflower oil (SAF oil) or Bio-C.L.A.

Diet		SAF Oil		Bio-C.L.A.		*p-*Values for Two-Way ANOVA		
	Group	SAF (n = 8)	SAFplus (n = 14)	CLA (n = 7)	CLAplus	Diet (D)	Mammary Tumors (MT)	Interaction (D × MT)
Variables					(n = 17)			
**CFA (μg/g)**		19.0 ± 4.19^ab^	13.8 ± 1.71^a^	74.1 ± 63.7^c^	32.0 ± 13.3^bc^	0.0002	0.0108	0.0429
**CD**		12.1 ± 2.79^a^	11.5 ± 2.35^a^	58.3 ± 51.1^b^	28.1 ± 11.1^b^	0.0001	0.0325	0.0393
*tt*		7.72 ± 1.82^a^	8.06 ± 1.18^a^	30.2 ± 26.9^b^	13.5 ± 5.54^b^	0.0006	0.0343	0.0278
*ct/tc*		2.86 ± 0.81	2.11 ± 0.55	25.6 ± 22.9	13.3 ± 5.52	0	0.0521	0.08
*cc*		1.03 ± 0.22^ab^	0.90 ± 0.27^a^	2.52 ± 1.64^b^	1.31 ± 0.47^ab^	0.0004	0.0096	0.0326
**CT**		6.90 ± 1.51^b^	2.81 ± 0.84^a^	15.7 ± 12.7^b^	3.26 ± 1.15^a^	0.0113	0	0.0211
*ttt*		4.55 ± 1.05^b^	1.02 ± 0.53^a^	11.7 ± 10.9^b^	1.12 ± 0.35^a^	0.0165	0	0.0194
*ttc*		0.20 ± 0.05	0.72 ± 0.61	0.22 ± 0.14	0.62 ± 0.44	0.78	0.0039	0.69
*cct*		2.10 ± 0.19^bc^	1.26 ± 0.22^a^	3.63 ± 1.51^c^	1.60 ± 0.62^ab^	0.0006	0	0.0218

Data are shown as mean values ± standard deviation (SD). *p-*value ≤ 0.05—significant differences among groups in the two-way ANOVA. 0.00—amount was below the quantification limit (<LOQ). D—diet; MT—mammary tumors, D × MT—interaction; When interaction (D × MT) occurs, the significance of differences among groups was further analyzed by a post hoc HSD Tukey test or multiple comparison tests. ^a, b, c^—values with different superscripts in rows significantly differ at *p-*value ≤ 0.05. SAF group—animals receiving safflower oil; SAFplus group—animals receiving safflower oil treated with DMBA; CLA group—group of animals receiving Bio-C.L.A.; CLAplus group—group of animals receiving Bio-C.L.A. treated with DMBA CFA—conjugated fatty acids, CD—conjugated dienes, CT—conjugated trienes, *cc*—*cis,cis* isomers*, ct/tc*—*cis,trans/ trans,cis* isomers, *tt*—*trans,trans* isomers, *ttt*—*trans,trans,trans* isomers, *ttc*—*trans,trans,cis* isomers, *cct*—*cis,cis,trans* isomers.

**Table 5:** Content of malondialdehyde (MDA), cholesterol and oxysterols in hearts of rats supplemented with safflower oil (SAF oil) or Bio-C.L.A.

Diet		SAF Oil		Bio-C.L.A.		*p-*Values for Two-Way ANOVA		
	Group	SAF (n = 8)	SAFplus (n = 14)	CLA (n = 7)	CLAplus (n = 17)	Diet (D)	Mammary Tumors (MT)	Interaction (D × MT)
Variables								
**MDA (ng/g)**		7182 ± 1018	8205 ± 1798	6171 ± 1548	6125 ± 1052	0.0012	0.28	0.24
**Cholesterol (mg/g)**		2.97 ± 0.10	3.05 ± 0.17	2.85 ± 0.15	2.95 ± 0.19	0.06	0.14	0.88
**Oxysterols**								
**7AOH (μg/g)**		0.94 ± 0.27	1.16 ± 0.51	0.79 ± 0.10	0.91 ± 0.27	0.12	0.19	0.68
**7BOH (μg/g)**		0.16 ± 0.08	0.14 ± 0.03	0.10 ± 0.01	0.14 ± 0.07	0.18	0.87	0.19
**5,6AE (μg/g)**		0.65 ± 0.17	0.80 ± 0.39	0.63 ± 0.08	0.66 ± 0.14	0.35	0.31	0.51
**7K (μg/g)**		1.65 ± 0.46	2.84 ± 1.41	1.38 ± 0.22	1.86 ± 0.64	0.06	0.0139	0.27

Data are shown as mean values ± standard deviation (SD). *p-*value ≤ 0.05—significant differences among groups in the two-way ANOVA. 0.00—amount was below the quantification limit (<LOQ). D—diet; MT—mammary tumors, D × MT—interaction; When interaction (D × MT) occurs, the significance of differences among groups was further analyzed by a post hoc HSD Tukey test or multiple comparison tests. ^a, b, c^—values with different superscripts in rows significantly differ at *p-*value ≤ 0.05. SAF group—animals receiving safflower oil; SAFplus group—animals receiving safflower oil treated with DMBA; CLA group—group of animals receiving Bio-C.L.A.; CLAplus group—group of animals receiving Bio-C.L.A. treated with DMBA. MDA—malondialdehyde; 7AOH—7α-hydroxycholesterol; 7BOH—7β-hydroxycholesterol; 5,6AE—cholesterol 5α,6α-epoxide; 7K—7-ketocholesterol.

## References

[1] Bray F., Ferlay J., Soerjomataram I., Siegel R.L., Torre L.A., Jemal A. (2018). Global cancer statistics 2018: GLOBOCAN estimates of incidence and mortality worldwide for 36 cancers in 185 countries. CA Cancer J. Clin..

[2] World Health Organization—WHO. http://www.who.int/cardiovascular_diseases/en/.

[3] Ferlay J., Soerjomataram I., Dikshit R., Eser S., Mathers C., Rebelo M., Parkin D.M., Forman D., Bray F. (2015). Cancer incidence and mortality worldwide: Sources, methods and major patterns in GLOBOCAN 2012. Int. J. Cancer.

[4] Koene R.J., Prizment A.E., Blaes A., Konety S. (2016). Shared Risk Factors in Cardiovascular Disease and Cancer. Circulation.

[5] Blaes A., Prizment A., Koene R.J., Konety S. (2017). Cardio-oncology Related to Heart Failure: Common Risk Factors Between Cancer and Cardiovascular Disease. Heart Fail. Clin..

[6] Liu V.Y., Agha A.M., Lopez-Mattei J., Palaskas N., Kim P., Thompson K., Mouhayar E., Marmagkiolis K., Hassan S.A., Karimzad K. (2018). Interventional Cardio-Oncology: Adding a New Dimension to the Cardio-Oncology Field. Front. Cardiovasc. Med..

[7] Venneri L., Caliccio F., Manivarmane R. (2015). Subclinical myocardial dysfunction in cancer patients: Is there a direct effect of tumour growth?. Eur. Heart J. Cardiovasc. Imaging.

[8] Asteggiano R., Suter T., Bax J.J. (2019). Cardio-oncology: Principles and organisational issues. E-Journal Cardiol. Pract..

[9] López-Fernandez T., Van der Meer P. (2019). Cardio-oncology: It is not only heart failure!. E-J. Cardiol. Pract..

[10] Marmot M., Atinmo T., Byers T., Chen J., Hirohata T., Jackson A., James W., Kolonel L., Kumanyika S., Leitzmann C. (2007). Food, Nutrition, Physical Activity, and the Prevention of Cancer: A Global Perspective.

[11] Mehta L.S., Watson K.E., Barac A., Beckie T.M., Bittner V., Cruz-Flores S., Dent S., Kondapalli L., Ky B., Okwuosa T. (2018). Cardiovascular Disease and Breast Cancer: Where These Entities Intersect: A Scientific Statement From the American Heart Association. Circulation.

[12] Patel A., Pathak Y., Patel J., Sutariya V. (2018). Role of nutritional factors in pathogenesis of cancer. Food Qual. Saf..

[13] Ferrini K., Ghelfi F., Mannucci R., Titta L. (2015). Lifestyle, nutrition and breast cancer: Facts and presumptions for consideration. Ecancermedicalscience.

[14] Pariza M.W., Ashoor S.H., Chu F.S., Lund D.B. (1979). Effects of temperature and time on mutagen formation in pan-fried hamburger. Cancer Lett..

[15] Pariza M.W., Loretz L.J., Storkson J.M., Holland N.C. (1983). Mutagens and modulator of mutagenesis in fried ground beef. Cancer Res..

[16] Pariza M.W., Hargraves W.A. (1985). A beef-derived mutagenesis modulator inhibits initiation of mouse epidermal tumors by 7,12-dimethylbenz[a]anthracene. Carcinogenesis.

[17] Białek A., Tokarz A. (2009). Źródła Pokarmowe Oraz Efekty Prozdrowotne Sprzężonych Dienów Kwasu Linolowego (CLA). Biul. Wydz. Farm. Warsz. Uniw. Med..

[18] Białek A., Zagrodzki P., Tokarz A. (2016). Chemometric analysis of the interactions among different parameters describing health conditions, breast cancer risk and fatty acids profile in serum of rats supplemented with conjugated linoleic acids. Prostaglandins Leukot. Essent. Fat. Acids.

[19] Zock P.L., Katan M.B. (1998). Linoleic acid intake and cancer risk. A review. Am. J. Clin Nutr..

[20] Białek A., Tokarz A., Zagrodzki P. (2014). Conjugated linoleic acids in diet of female rats inhibit the breast cancer formation in their offspring. J. Food Nutr. Res..

[21] Białek A., Tokarz A., Zagrodzki P. (2015). Conjugated Linoleic Acids (CLA) Decrease the Breast Cancer Risk in DMBA-Treated Rats. Acta Pol. Pharm..

[22] Kelley D.S., Bartolini G.L., Newman J.W., Vemuri M., Mackey B.E. (2006). Fatty acid composition of liver, adipose tissue, spleen, and heart of mice fed diets containing t10, c12-, and c9, t11-conjugated linoleic acid. Prostaglandins Leukot. Essent. Fat. Acids.

[23] Yuan G.-F., Sinclair A.J., Sun H.-Y., Li D. (2009). Fatty Acid Composition in Tissues of Mice Fed Diets Containing Conjugated Linolenic Acid and Conjugated Linoleic Acid. J. Food Lipids.

[24] Alasnier C., Berdeaux O., Chardigny J.M., Sébédio J.L. (2002). Fatty acid composition and conjugated linoleic acid content of different tissues in rats fed individual conjugated linoleic acid isomers given as triacylglycerols. J. Nutr. Biochem..

[25] Diniz Y.S., Santos P.P., Assalin H.B., Souza G.A., Rocha K.K.H.R., Ebaid G.M.X., Seiva F.R.F., Amauchi J.F., Novelli Filho J.L.V.B., Novelli E.L.B. (2008). Conjugated linoleic acid and cardiac health: Oxidative stress and energetic metabolism in standard and sucrose-rich diets. Eur. J. Pharmacol..

[26] Arab L. (2003). Biomarkers of fat and fatty acid intake. J. Nutr..

[27] Czauderna M., Kowalczyk J., Korniluk K., Wasowska I. (2007). Improved saponification then mild base and acid-catalyzed methylation is a useful method for quantifying fatty acids, with special emphasis on conjugated dienes. Acta Chromatogr..

[28] Białek M., Czauderna M., Białek A. (2018). Partial replacement of rapeseed oil with fish oil, and dietary antioxidants supplementation affects concentrations of biohydrogenation products and conjugated fatty acids in rumen and selected lamb tissues. Anim. Feed Sci. Technol..

[29] Lopes L.D., Böger B.R., Cavalli K.F., dos S., Silveira-Júnior J.F., Osório D.V.C.L., de Oliveira D.F., Luchetta L., Tonial I.B. (2014). Fatty acid profile, quality lipid index and bioactive compounds of flour from grape residues. Cienc. e Investig. Agrar..

[30] Ghaeni M., Ghahfarokhi K.N. (2013). Fatty Acids Profile, Atherogenic (IA) and Thrombogenic (IT) Health Lipid Indices in Leiognathusbindus and Upeneussulphureus. J. Mar. Sci. Res. Dev..

[31] Bialek A., Bialek M., Jelinska M., Tokarz A. (2017). Fatty acid composition and oxidative characteristics of novel edible oils in Poland. CyTA-Food.

[32] Czauderna M., Marounek M., Duskova D., Kowalczyk J. (2013). The sensitive and simple measurement of underivatized cholesterol and its oxygen derivatives in biological materials by capillary gas chromatography coupled to a mass-selective detector. Acta Chromatogr..

[33] Czauderna M., Kowalczyk J., Marounek M. (2011). The simple and sensitive measurement of malondialdehyde in selected specimens of biological origin and some feed by reversed phase high performance liquid chromatography. J. Chromatogr. B Anal. Technol. Biomed. Life Sci..

[34] (2016). Statistica Data Analysis Software System, version 13.

[35] Anand P., Kunnumakara A.B., Sundaram C., Harikumar K.B., Tharakan S.T., Lai O.S., Sung B., Aggarwal B.B. (2008). Cancer is a preventable disease that requires major lifestyle changes. Pharm. Res..

[36] Adami H.-O., Day N.E., Trichopoulos D., Willett W.C. (2001). Primary and secondary prevention in the reduction of cancer morbidity and mortality. Eur. J. Cancer.

[37] S.717—21st Century Cancer ALERT (Access to Life-Saving Early detection, Research and Treatment) Act. https://www.congress.gov/bill/111th-congress/senate-bill/717/text.

[38] Bougnoux P., Hajjaji N., Maheo K., Couet C., Chevalier S. (2010). Fatty acids and breast cancer: Sensitization to treatments and prevention of metastatic re-growth. Prog. Lipid Res..

[39] Tappia P.S., Mangat R., Gabriel C., Dent M.R., Aroutiounova N., Weiler H. (2006). Gender differences in the cardiac response to dietary conjugated linoleic acid isomers. Can. J. Physiol. Pharmacol..

[40] Kuo C.Y., Ann D.K. (2018). When fats commit crimes: Fatty acid metabolism, cancer stemness and therapeutic resistance. Cancer Commun..

[41] Baenke F., Peck B., Miess H., Schulze A. (2013). Hooked on fat: The role of lipid synthesis in cancer metabolism and tumour development. Dis. Model. Mech..

[42] Ayalew-Pervanchon A., Rousseau D., Moreau D., Assayag P., Weill P., Grynberg A. (2007). Long-term effect of dietary α-linolenic acid or decosahexaenoic acid on incorporation of decosahexaenoic acid in membranes and its influence on rat heart in vivo. Am. J. Physiol. Circ. Physiol..

[43] Brochot A., Guinot M., Auchere D., MacAire J.P., Weill P., Grynberg A. (2009). Effects of alpha-linolenic acid vs. docosahexaenoic acid supply on the distribution of fatty acids among the rat cardiac subcellular membranes after a short- or long-term dietary exposure. Nutr. Metab..

[44] Van Bilsen M., Planavila A. (2014). Fatty acids and cardiac disease: Fuel carrying a message. Acta Physiol..

[45] Białek A., Stawarska A., Tokarz A., Czuba K., Konarska A., Mazurkiewicz M., Stanimirova-Daszykowska I. (2014). Enrichment of maternal diet with conjugated linoleic acids influences desaturases activity and fatty acids profile in livers and hepatic microsomes of the offspring with 7,12-dimethylbenz[A]anthracene-induced mammary tumors. Acta Pol. Pharm-Drug Res..

[46] Tsuzuki T., Ikeda I. (2007). Slow Absorption of Conjugated Linoleic Acid in Rat Intestines, and Similar Absorption Rates of 9 c,11 t -Conjugated Linoleic Acid and 10 t,12 c -Conjugated Linoleic Acid. Biosci. Biotechnol. Biochem..

[47] Chaplin A., Parra P., Serra F., Palou A. (2015). Conjugated Linoleic Acid Supplementation under a High-Fat Diet Modulates Stomach Protein Expression and Intestinal Microbiota in Adult Mice. PLoS ONE.

[48] Kamlage B., Hartmann L., Gruhl B., Blaut M. (1999). Intestinal microorganisms do not supply associated gnotobiotic rats with conjugated linoleic acid. J. Nutr..

[49] Jelińska M., Białek A., Gielecińska I., Mojska H., Tokarz A. (2017). Impact of conjugated linoleic acid administered to rats prior and after carcinogenic agent on arachidonic and linoleic acid metabolites in serum and tumors. Prostaglandins Leukot. Essent. Fat. Acids.

[50] Gonenc A., Ozkan Y., Torun M., Simsek B. (2003). Plasma malondialdehyde (MDA) levels in breast and lung cancer patients. J. Clin. Pharm. Ther..

[51] Czauderna M., Kowalczyk J., Wąsowska I., Niedźwiedzka K., Pastuszewska B. (2003). The effects of selenium and conjugated linoleic acid (CLA) isomers on fatty acid composition, CLA isomer content in tissues, and growth of rats. J. Anim. Feed Sci..

[52] Khosla P., Fungwe T.V. (2001). Conjugated linoleic acid: Effects on plasma lipids and cardiovascular function. Curr. Opin. Lipidol..

[53] Mutemberezi V., Guillemot-Legris O., Muccioli G.G. (2016). Oxysterols: From cholesterol metabolites to key mediators. Prog. Lipid Res..

[54] Sottero B., Leonarduzzi G., Testa G., Gargiulo S., Poli G., Biasi F. (2019). Lipid Oxidation Derived Aldehydes and Oxysterols Between Health and Disease. Eur. J. Lipid Sci. Technol..

[55] Kulig W., Cwiklik L., Jurkiewicz P., Rog T., Vattulainen I. (2016). Cholesterol oxidation products and their biological importance. Chem. Phys. Lipids.

[56] Brzeska M., Szymczyk K., Szterk A. (2016). Current Knowledge about Oxysterols: A Review. J. Food Sci..

[57] Grandgirard A., Demaison-Meloche J.C.C., Demaison L. (2004). Incorporation of oxyphytosterols in tissues of hamster. Reprod. Nutr. Dev..

[58] Adachi J., Kudo R., Ueno Y., Hunter R., Rajendram R., Want E., Preedy V.R. (2001). Heart 7-Hydroperoxycholesterol and Oxysterols Are Elevated in Chronically Ethanol-Fed Rats. J. Nutr..

[59] Combe N., Clouet P., Chardigny J.M., Lagarde M., Léger C.L. (2007). Trans fatty acids, conjugated linoleic acids, and cardiovascular diseases. Eur. J. Lipid Sci. Technol..

[60] Wannamethee S.G., Jefferis B.J., Lennon L., Papacosta O., Whincup P.H., Hingorani A.D. (2018). Serum conjugated linoleic acid and risk of incident heart failure in older men: The British regional heart study. J. Am. Heart Assoc..

